# Supplementary material for: Fusion of fully integrated analog machine learning classifier with electronic medical records for real-time prediction of sepsis onset
Source: Sci Rep. 2022 Apr 5;12:5711. doi: 10.1038/s41598-022-09712-w (PMC8983688; doi:10.1038/s41598-022-09712-w)
Supplement: Supplementary file 1 — Supplementary Information 1. [file 41598_2022_9712_MOESM1_ESM.pdf]

Fusion of Fully Integrated Analog Machine Learning Classifier with Electronic Medical Records for Real-time Prediction of Sepsis Onset: Supplementary Information

Sudarsan Sadasivuni[1,+], Monjoy Saha[2,+], Neal Bhatia[4], Imon Banerjee[2,3,++], and Arindam Sanyal[1,*,++]

[1]University at Buffalo, Electrical Engineering, Buffalo, 14260, USA
[2]Emory University, Department of Biomedical Informatics, Atlanta, 30322, USA
[3]Emory University, Department of Radiology, Atlanta, 30322, USA
[4]Emory University School of Medicine, Division of Cardiology, Atlanta, 30322, USA
[*]arindams@buffalo.edu
[+]these authors contributed equally to this work
[++]co-senior authors

Additional performance Analysis

Baseline EMR model - Figure 1 present the ROC curve for the baseline EMR model which achieved 0.79 AUROC value.

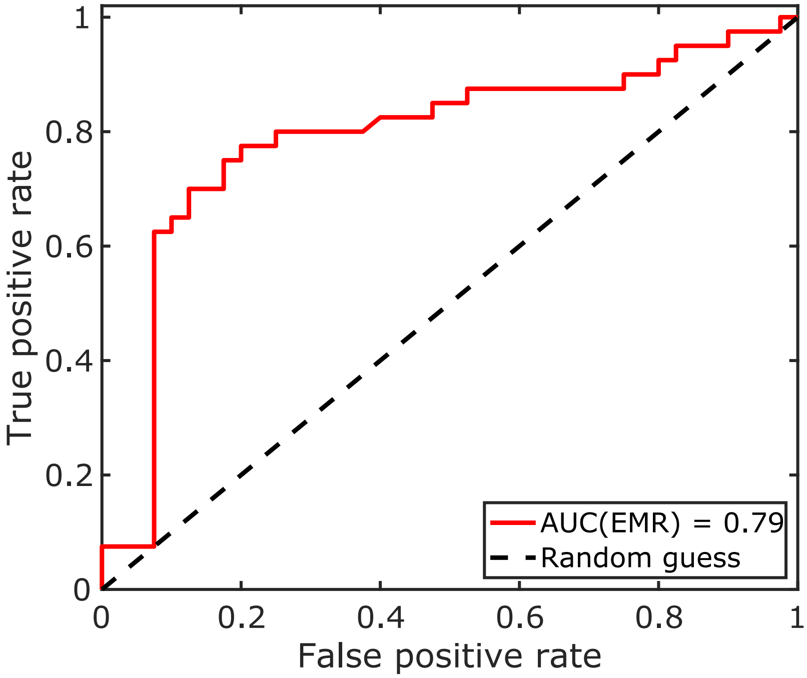

Figure 1. Receiver operating characteristic curve of the baseline EMR model - using demographics and co-morbidity data

Model selection using 10-fold cross-validation - Figures 2 show visual comparison of different classifier architectures for late fusion. The box plots shows the accuracy of different classifiers at different time-internals along with error bar for 10-fold cross-validation.

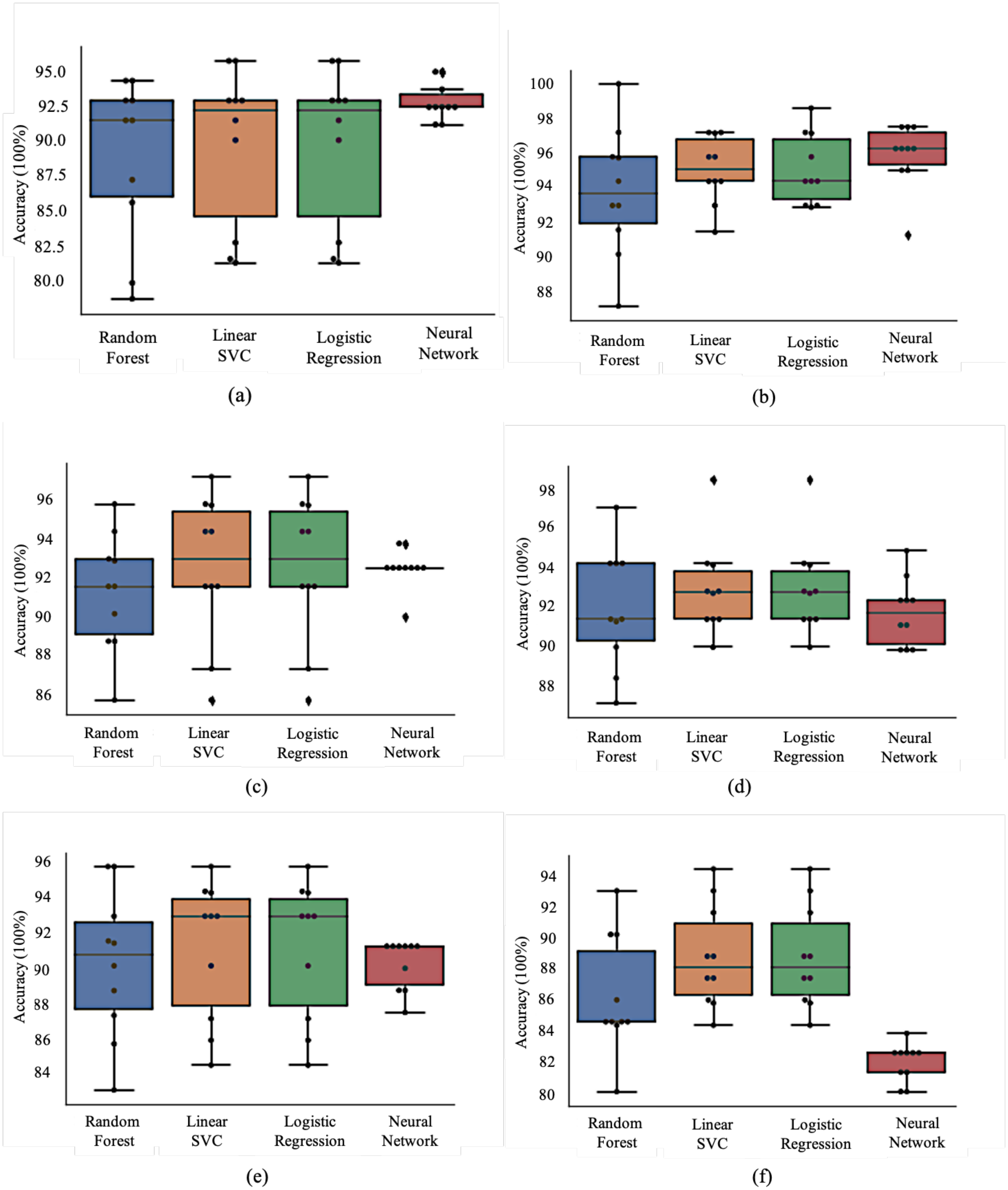

Figure 2. Box plots for late fusion performance analysis of different classifiers using demographic, co-morbidity and ECG data; (a) 1 hr. data; (b) 2 hrs. data ; (c) 3 hrs. data; (d) 4 hrs. data; (e) 5 hrs. data; (f) 6 hrs. data

Confusion matrix for late fusion model - Figures 3 shows late fusion confusion matrices (2x2) for each time point where each cell represents the patient counts. The confusion matrices show the true positive and true positive values along the diagonals.

Comparison with analog in-memory computation macros using SRAM

Aside from using switched-capacitor MAC circuits for analog IMC, several works re-use static random access memory (SRAM) array that holds ANN weights for analog IMC[1–6]. Figure 4 compares the two analog IMC techniques. Compared to SRAM array (see the Supplement), the switched-capacitor IMC adopted in this work has two advantages - 1) higher linearity, 2) better matching. Multiplication is performed in SRAM cell by applying analog input to the wordline (WL) which draws a proportional current, I_{ds} from the differential readlines (BL and BLB). The current I_{ds} discharges voltage on BL/BLB lines, and accumulation is performed in charge-domain on the BL/BLB lines. The in-memory vector matrix multiplication (VMM) is linear as long as I_{ds} is linearly proportional to the voltage applied on the WL line, and is independent of the accumulated voltage on the BL/BLB lines. However, for large values of VMM output, the transistor drawing I_{ds} is pushed into triode region, and I_{ds} becomes a nonlinear function of the voltage on BL/BLB lines, thus making the VMM result nonlinear. This is a fundamental limitation of SRAM based IMC techniques. In contrast, the switched capacitor IMC performs VMM through passive charge redistribution between the capacitors in the array which makes the VMM computation highly linear. Random mismatches during chip fabrication process introduces random variations into each circuit component, and hence, ANN weights which makes VMM results inaccurate. However, it is easier to match passive components, like capacitors, with high accuracy than transistors. Since switched-capacitor IMCs compute VMM results based on ratios of capacitors, it is more accurate than SRAM IMC.

0.1 Label encoding versus one-hot encoding for EMR model

The categorical and textual EMR data need to be converted into numeric form for analysis with EMR model. Label encoding and one-hot encoding are two popular encoding techniques for conversion of categorical/textual data into numeric format. However, there are trade-offs involved when using these encoding techniques. Label encoding imposes ordinality to categorical data, while one-hot encoding increases dimensionality of categorical data. We use label encoding in our work because of two reasons – a) the EMR model uses random forest classifier which has a much better performance than other models considered (linear SVM, logistic regression, artificial neural network). Random forest can directly accept categorical variables and often perform better with label encoding than one-hot encoding. One-hot encoding introduces undesirable sparsity to the data since the one-hot encoded columns are mostly zeros, and tree-based models, such as random forest, will assign low importance to the one-hot encoded columns since splitting on them will only produce a small gain b) the categorical variables in our work has low number of levels (7 for race and 3 or lower for the other categorical variables) and hence, performance of the models do not change much going from label encoding to one-hot encoding. This is shown in Table 1 which shows that there is little difference in performance of the models for categorical encoding and one-hot encoding.

Table 1. Comparison of label and one-hot encoding for EMR model. Optimal performance for every prediction task is highlighted in **bold**.

	Label encoding		One-hot encoding	
	Accuracy (%)	AUROC	Accuracy (%)	AUROC
Linear SVM	49	0.47	51	0.54
Logistic Regression	53	0.54	52	0.51
Random Forest	**76**	**0.79**	**76**	0.78
Neural Network	51	0.50	51	0.50

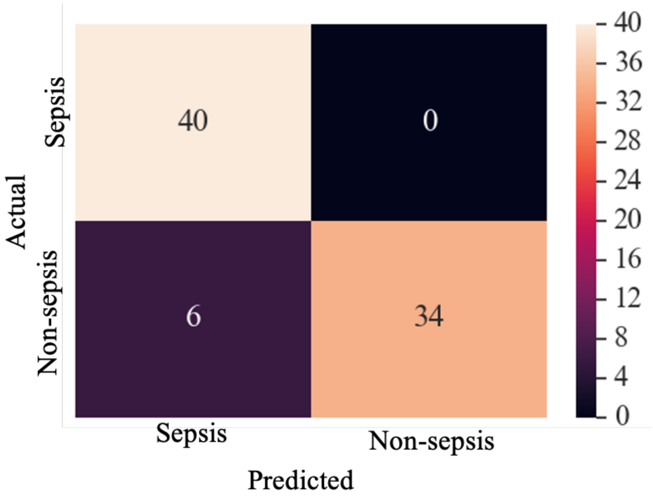

(a)

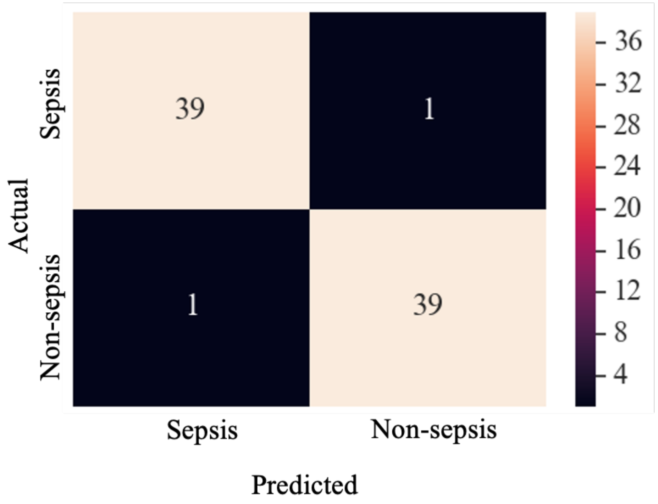

(b)

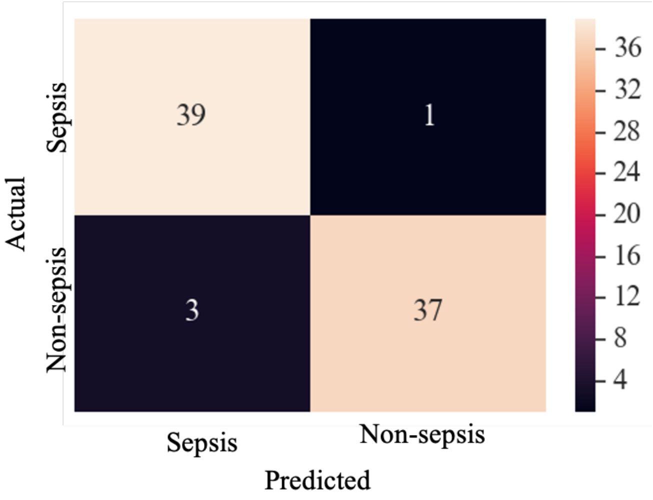

(c)

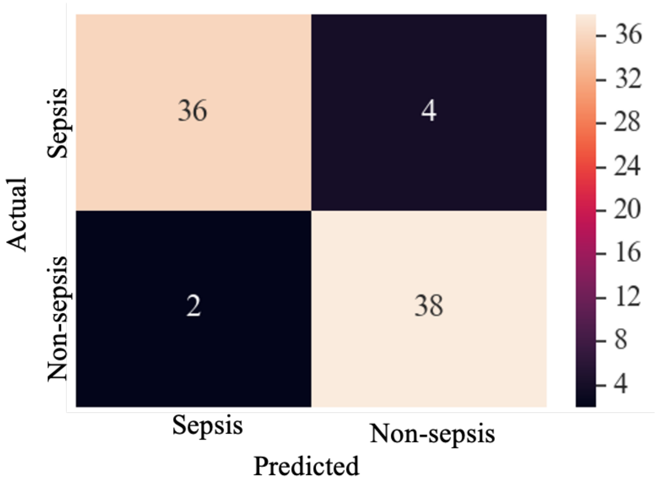

(d)

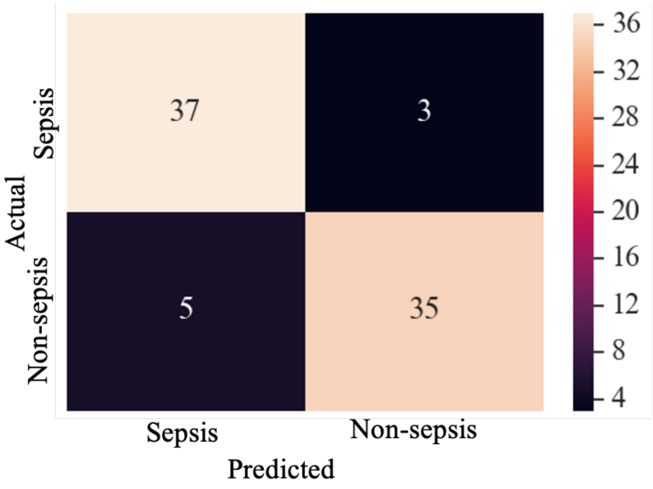

(e)

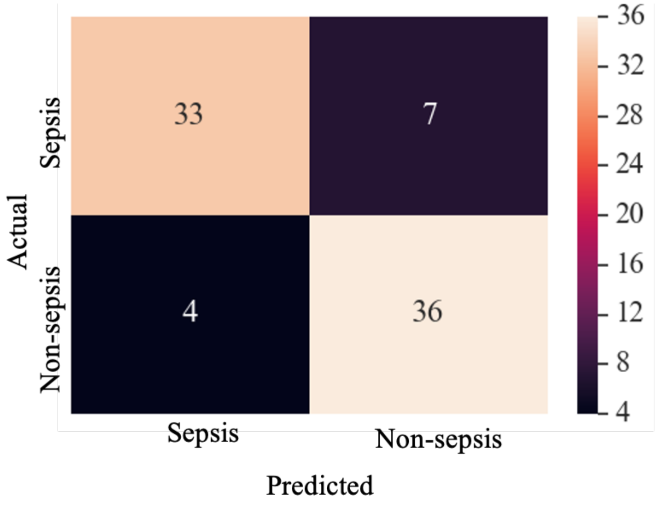

(f)

Figure 3. Confusion matrix for late fusion using demographic, co-morbidity, and ECG data for different sepsis on-set prediction tasks; (a) 1 hr; (b) 2 hrs; (c) 3 hrs; (d) 4 hrs; (e) 5 hrs; (f) 6 hrs. Only optimal prediction results are shown.

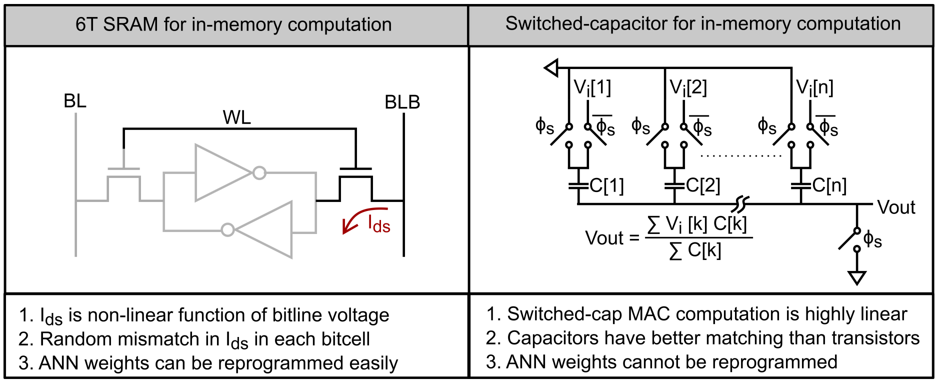

Figure 4. Comparison with analog in-memory computation using SRAM cells

References

1. Zhang, J., Wang, Z. & Verma, N. A machine-learning classifier implemented in a standard 6T SRAM array. In *IEEE Symposium on VLSI Circuits (VLSI-Circuits)*, 1–2 (2016).
2. Valavi, H., Ramadge, P. J., Nestler, E. & Verma, N. A mixed-signal binarized convolutional-neural-network accelerator integrating dense weight storage and multiplication for reduced data movement. In *IEEE Symposium on VLSI Circuits*, 141–142 (2018).
3. Biswas, A. & Chandrakasan, A. P. Conv-RAM: An energy-efficient SRAM with embedded convolution computation for low-power CNN-based machine learning applications. In *IEEE ISSCC*, 488–490 (2018).
4. Dong, Q. *et al.* A 351TOPS/W and 372.4 GOPS compute-in-memory SRAM macro in 7nm FinFET CMOS for machine-learning applications. In *2020 IEEE International Solid-State Circuits Conference-(ISSCC)*, 242–244 (IEEE, 2020).
5. Gonugondla, S. K., Kang, M. & Shanbhag, N. A 42pj/decision 3.12 tops/w robust in-memory machine learning classifier with on-chip training. In *2018 IEEE International Solid-State Circuits Conference-(ISSCC)*, 490–492 (IEEE, 2018).
6. Si, X. *et al.* 24.5 a twin-8t sram computation-in-memory macro for multiple-bit cnn-based machine learning. In *2019 IEEE International Solid-State Circuits Conference-(ISSCC)*, 396–398 (IEEE, 2019).
